# The Role of Duration of Untreated Illness (DUI) on the Course and Biochemical Parameters of Female Patients Affected by Anorexia Nervosa

**DOI:** 10.1002/brb3.70584

**Published:** 2025-05-30

**Authors:** Letizia Maria Affaticati, Enrico Capuzzi, Tommaso Zuliani, Francesca Legnani, Nadia Vaccaro, Francesca Manzo, Alberto Scalia, Davide La Tegola, Monica Nicastro, Fabrizia Colmegna, Massimo Clerici, Antonios Dakanalis, Massimiliano Buoli

**Affiliations:** ^1^ Department of Medicine and Surgery University of Milan Bicocca Monza Italy; ^2^ Department of Mental Health Fondazione IRCCS San Gerardo dei Tintori Monza Italy; ^3^ University of Milan Milan Italy; ^4^ Department of Neurosciences and Mental Health Fondazione IRCCS Ca'Granda Ospedale Maggiore Policlinico Milan Italy; ^5^ Department of Pathophysiology and Transplantation University of Milan Milan Italy

**Keywords:** anorexia nervosa, duration of untreated illness (DUI), clinical variables, biochemical parameters

## Abstract

**Objective:**

Anorexia nervosa (AN) presents the highest rate of mortality among psychiatric disorders, making an early diagnosis and proper management essential. The purpose of the study was to investigate the role of duration of untreated illness (DUI) on the course and biochemical parameters in a sample of 76 female patients with AN.

**Method:**

Correlation analyses and one‐way analyses of variance were performed to analyze the relation between DUI and quantitative and qualitative variables, respectively. Statistically significant factors from correlation analyses were inserted in a linear regression model as independent variables with the DUI as the dependent variable.

**Results:**

A longer DUI was directly correlated with age (*r* = 0.35, *p* < 0.01), duration of illness (*r* = 0.61, *p* < 0.01), potassium (K) plasma levels (*r* = 0.47, *p* = 0.02), and platelet/lymphocyte ratio (PLR) (*r* = 0.25, *p* = 0.04). A longer DUI was inversely correlated with the sodium/potassium ratio (Na/K) (*r* = −0.42, *p* = 0.047). Linear regression analysis confirmed the direct association between a longer DUI with age (*B* = 0.12, *p* = 0.01) and K levels (*B* = 3.18, *p* = 0.017).

**Discussion:**

The higher K plasma levels in patients with longer DUI may indicate an abuse of supplements and/or an alteration of the hypothalamus–pituitary–axis (HPA) or renin–angiotensin systems. In support of this hypothesis, alterations in PLR were identified in subjects with abnormal bone mineral density. Further research is needed to confirm the present findings.

## Graphical abstract

We studied the impact of duration of untreated illness (DUI) on the course of 76 patients affected by anorexia nervosa. Our findings showed that patients with longer DUIs have specific clinical and biochemical alterations. Prevention strategies are necessary to reduce DUI and improve AN prognosis.

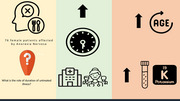

## 1 Introduction

Anorexia nervosa (AN) is a prevalent psychiatric condition characterized by the fear of weight gain, disturbances in self‐perceived weight or shape, and behaviors leading to weight loss (Yu and Muehleman 2023). This disorder is associated with poor quality of life (van Hoeken and Hoek 2020) and at least five times more elevated mortality compared to healthy subjects (van Eeden et al. 2021). Different clinical factors were identified to contribute to a poor course of AN, including comorbidity with anxiety, low body mass index (BMI), and duration of illness (Glasofer et al. 2020). Furthermore, a study highlighted that a later age at onset was correlated with a lower BMI at the time of the patients’ first medical examination (Kimura et al. 2007).

National and international guidelines recommend psychotherapy and specifically Cognitive Behavioral Therapy (CBT) as first‐line treatment for adults with AN (Resmark et al. 2019). With regard to pharmacotherapy, only olanzapine has limited evidence and is restricted to weight gain (Himmerich et al. 2023). A proper treatment for this condition should be rapidly planned for the negative consequences on patients’ mental and physical health, including cognitive deficits or more susceptibility to infections (Zipfel et al. 2015). On the other hand, a longer duration of untreated illness (DUI), defined as the time between the onset of the disorder and the prescription of a proper treatment (Esposito et al. 2023), was repeatedly reported as a factor that is negatively associated with the course of most psychiatric conditions, including schizophrenia (Murru and Carpiniello 2018), mood (Luo et al. 2023), and anxiety disorders (Surace et al. 2024).

Despite these aspects, little data has been published to date about the effects of delayed diagnosis and treatment in subjects affected by AN. In a 20‐year follow‐up study, a longer DUI was found to be associated with an increased risk of a current eating disorder in a sample of 62 women diagnosed as affected by AN in adolescence (Andrés‐Pepiñá et al. 2020). Other authors remarked on the importance of the primary care practitioners as key figures to reduce DUI length (Gumz et al. 2023). Another factor that can influence DUI is age at onset: Neubauer et al. (2014) highlighted that individuals with an earlier age at onset (≤ 14 years old) were less motivated to seek medical help, needing the caregivers’ support.

In light of the paucity of data on this topic, the purpose of the present data is to identify the clinical factors and the biochemical parameters associated with DUI in female patients affected by AN.

## 2 Method

A total of 76 women affected by AN were recruited for the purposes of this study. The patients were selected among those who had been hospitalized at the Inpatient Clinic of Fondazione IRCCS Ca' Granda, Ospedale Maggiore Policlinico (Milan), or had been followed up at the Outpatient Clinic for Eating Disorders of Fondazione IRCCS San Gerardo dei Tintori (Monza). Study procedures were approved by the Ethics Committee of San Gerardo Hospital (Monza) (protocol number 3951).

Inclusion criteria consisted of (1) a diagnosis of AN according to the Diagnostic and Statistical Manual of Mental Disorders (DSM), fifth edition (DSM‐5) (American Psychiatric Association 2013), and (2) an age of ≥ 17 years. Exclusion criteria included (1) malnourishment due to severe organic disease; (2) pregnancy or breastfeeding, as the perinatal period is associated with an increased risk of mood and anxiety disorders in women (Gopalan et al. 2022); and (3) taking vitamin B or vitamin D supplements.

The following sociodemographic and clinical variables were collected during the first psychiatric examination: age, marital status, occupation status, age at onset, duration of illness, DUI, BMI, education (years), single or multiple substance use disorders, family history of psychiatric disorders, current poly‐pharmacotherapy, current psychotherapy, psychiatric comorbidity, multiple psychiatric comorbidity, medical comorbidity, multiple medical comorbidity, and personality disorders.

DUI was defined as the time period elapsed between AN onset and the first appropriate treatment according to international guidelines (Buoli et al. 2021; Resmark et al. 2019). This is the generally accepted definition of DUI for various psychiatric disorders (Perris et al. 2023), although a previous study on AN defined DUI as the difference between the date of onset of AN and the date of first treatment initiation, regardless of the appropriateness of the treatment (Gumz et al. 2023). In case of psychiatric comorbidity, AN represented the disorder more associated with social dysfunction.

Blood samples were collected fasting in the morning during the first visit at the outpatient clinic or at the beginning of hospitalization at the inpatient clinic. The following peripheral biological parameters were assessed: total number of red blood cells (10^6^/mm^3^), mean corpuscular volume (fL), hemoglobin (g/dL), total number of white blood cells (10^9^/L), lymphocytes (10^9^/L), neutrophils (10^9^/L), platelets (10^9^/L), plasma levels of sodium (Na) (mmol/L), potassium (K), iron (µg/dL), folic acid (ng/mL), vitamin D (ng/mL), vitamin B12 (pg/mL), and total cholesterol (mg/dL). NLR and PLR were then calculated by dividing, respectively, the total neutrophil count and the total platelet count by the total number of lymphocytes. Finally, the Na/K ratio was also calculated.

Descriptive analyses on the total sample were performed. DUI was treated as a continuous variable. Correlation analyses were performed between DUI and continuous variables. Groups identified by qualitative variables were compared in terms of DUI by one‐way analyses of variance. The variables that were statistically significant at univariate analyses were then inserted as independent variables in a linear regression model with DUI as the dependent variable. The Durbin–Watson test was used to verify the reliability of the model.

Statistical analyses were performed through the Statistical Package for Social Sciences (SPSS) for Windows (version 28.0). The level of statistical significance was set at *p *≤ 0.05.

## 3 Results

The total sample consisted of 76 women. Among them, 48 did not receive any pharmacological treatment, while 21 were treated with antidepressants, 6 with atypical antipsychotics, and 1 with typical antipsychotics. A total of 25 patients (32.9%) had lifetime psychiatric comorbidity: 10 major depressive disorder, 8 anxiety disorders, 4 personality disorders, and 3 obsessive–compulsive disorder. The results of descriptive and correlation analyses, as well as the values of DUI according to qualitative variables, are reported in Table 1 (sociodemographic and clinical variables) and Table 2 (biochemical parameters).

**TABLE 1:** Sociodemographic and clinical variables of the total sample and values of DUI/Pearson's correlation according to these variables.

Variables		Total sample, *N* = 76	DUI (mean ± SD)	*p*
Marriage or partnership Missing *n* = 10	Yes	19 (28.8%)	1.92 ± 2.59	0.73
	No	47 (71.2%)	2.19 ± 2.94	
Occupational status Missing *n* = 3	Employed	11 (15.1%)	3.51 ± 5.20	0.19
	Unemployed	13 (17.8%)	2.00 ± 2.12	
	Student	49 (67.1%)	1.85 ± 1.99	
Lifetime substance use disorders Missing *n* = 2	Yes	9 (12.2%)	1.77 ± 0.65	0.70
	No	65 (87.8%)	2.89 ± 0.36	
Lifetime poly‐substance use disorders Missing *n* = 3	Yes	3 (4.1%)	1.33 ± 0.58	0.62
	No	70 (95.9%)	2.13 ± 2.79	
Family history of psychiatric disorders Missing *n* = 2	Yes	25 (33.8%)	2.70 ± 0.54	0.22
	No	49 (66.2%)	2.72 ± 0.39	
Current poly‐pharmacotherapy Missing *n* = 14	Yes	12 (19.4%)	2.67 ± 3.06	0.62
	No	50 (80.6%)	2.19 ± 2.91	
Current psychotherapy Missing *n* = 14	Yes	4 (6.5%)	2.50 ± 3.70	0.88
	No	58 (93.5%)	2.27 ± 2.90	
Psychiatric comorbidity	Yes	25 (32.9%)	2.17 ± 2.35	0.88
	No	51 (67.1%)	2.08 ± 2.86	
Multiple psychiatric comorbidity	Yes	8 (10.5%)	2.75 ± 2.96	0.48
	No	68 (89.5%)	2.03 ± 2.66	
Medical comorbidity	Yes	20 (27.0%)	2.47 ± 3.66	0.52
	No	54 (73.0%)	2.00 ± 2.30	
Multiple medical comorbidity	Yes	3 (4.2%)	2.34 ± 3.21	0.90
	No	69 (95.8%)	2.14 ± 2.76	
Personality disorder	Yes	13 (17.1%)	3.08 ± 4.54	0.15
	No	63 (82.9%)	1.91 ± 2.12	
**Variables**		**Total sample, *N* = 76**	**Pearson's correlation (*r*)**	***p***
Age		23.12 ± 8.32	0.35	**< 0.01**
Education (years)		12.21 ± 3.04	0.12	0.33
Age at onset (years)		18.42 ± 6.48	−0.04	0.71
Duration of illness (years)		4.17± 5.81	0.61	**< 0.01**
BMI (kg/m^2^)		17.23 ± 2.23	−0.10	0.38

*Note*: In bold, statistically significant p for the following analyses: analyses of variance for qualitative variables and correlation analysis for continuous ones. Means ± standard deviations of the total sample are reported for continuous variables. The percentages referred to the total sample (excluding the missing data) are reported into brackets for qualitative variables.

Abbreviations: BMI, body mass index; DUI, duration of untreated illness.

**TABLE 2:** Biochemical parameters of the total sample and values of Pearson's correlation according to these variables.

**Variables**	**Total sample, *N* = 76**	**Pearson's correlation (*r*)**	***p***
Na (mmol/L)	140.33 ± 2.78	−0.34	0.10
K (mmol/L)	4.33 ± 0.35	0.47	**0.02**
Na/K	32.66 ± 2.68	−0.42	**0.04**
Red blood cells (10^6^/mm^3^)	4.27 ± 0.44	−0.15	0.46
MCV (fL)	90.82 ± 5.47	−0.22	0.30
Hb (g/dL)	13.05 ± 1.26	−0.29	0.14
White blood cells (10^9^/L)	5.67 ± 1.76	−0.14	0.25
Number of lymphocytes (10^9^/L)	2.13 ± 0.75	−0.15	0.20
Number of neutrophils (10^9^/L)	3.23 ± 1.70	−0.02	0.90
Platelets (10^9^/L)	227.66 ± 49.34	0.09	0.46
NLR	1.55 ± 0.94	0.07	0.63
PLR	117.06 ± 41.06	0.25	**0.04**
Iron (µg/dL)	106.60 ± 74.72	−0.07	0.62
Vitamin D (ng/mL)	30.58 ± 14.24	−0.09	0.59
Folate (ng/mL)	8.86 ± 6.24	0.16	0.24
Vitamin B12 (pg/mL)	529.43 ± 325.68	0.14	0.31
Total cholesterol (mg/dL)	172.10 ± 34.94	−0.07	0.62

*Note*: In bold, statistically significant *p*. Means ± standard deviations of the total sample are reported.

Abbreviations: BMI, body mass index; Hb, hemoglobin; K, potassium; MCV, mean corpuscular volume; Na, sodium; NLR, neutrophil/lymphocyte ratio; PLR, platelet/lymphocyte ratio.

A longer DUI was significantly correlated with age (*r* = 0.35, *p* < 0.01), duration of illness (*p* < 0.01), K plasma levels (*r* = 0.47, *p* = 0.02), Na/K (*r* = −0.42, *p* = 0.04), and PLR (*r* = 0.25, *p* = 0.04).

The linear regression model was found to be reliable (Durbin–Watson's test: 2.30). DUI resulted to be significantly associated with K plasma levels (*B* = 3.18, *p* = 0.02) and age (*B* = 0.12, *p* = 0.01).

## 4 Discussion

This was one of the few studies that evaluated the impact of a delayed diagnosis on clinical aspects and biochemical parameters in women affected by AN. DUI was found to be significantly related to age, K plasma levels, PLR, Na/K, and duration of illness.

With regard to the association between DUI and age/duration of illness, it is possible to make the hypothesis that a part of women with AN can escape medical attention and receive proper treatment only at a certain age. Factors that were identified to hamper continuous follow‐up and treatment include specific clinical features such as prominent anger–hostility (Todisco et al. 2023) or the binge‐purging subtype of AN (Fassino et al. 2009). A recent review identified the barriers of early intervention in AN: as well as motivational factors (poor insight or stigma), the authors highlighted that patients often have to face long waiting lists and limited treatment options (Mills et al. 2023). In this sense, prevention and treatment services for AN should be implemented also at the primary care level (Klein et al. 2021).

The association between K plasma levels and DUI could be explained by the abuse of dietary supplements by women suffering from chronic anorexia. This is consistent with the fact that patients with AN engage in extreme sports practices associated with the abuse of supplements aimed at improving sports performance to increase calorie consumption (Savino et al. 2019). Furthermore, electrolyte alterations involving Na or K could be an indirect indicator of anomalies in the hypothalamus–pituitary–axis (HPA) or in the renin–angiotensin–aldosterone system that are well documented in women suffering from AN and probably more marked in those who escape medical care for a longer time (Mizuno et al. 1993). An alternative explanation can be that hyperkalemia is due to compensatory mechanisms of chronic malnutrition. Of note, patients with long‐term AN often suffer from renal failure, which results in several metabolic alterations, including abnormal potassium levels (Puckett 2023).

No data are available regarding the correlation between PLR and DUI in AN; however, a study demonstrated an association between PLR and a lower bone mineral density (Morawiecka‐Pietrzak et al. 2021). PLR is also a marker of inflammation, and therefore, an increase of this parameter in patients with a longer DUI is an indicator of more dysfunction of biological systems (Wei et al. 2022). Of note, PLR was found to be higher than healthy controls in a number of psychiatric conditions, including mood disorders (Marazziti et al. 2022) and schizophrenia (Özdin and Böke 2019). It should be noted that bone metabolism involves several factors and that some studies revealed a negative correlation between vitamin D and PLR (Akbas et al. 2016). Vitamin D deficiency, in turn, was associated with worse clinical features, including cognitive dysfunction in subjects with AN (Todisco et al. 2024).

Taken as a whole, a longer DUI would characterize women with an older age who have potentially escaped medical attention, with abuse of supplements or with a dysregulation of the hypothalamic–pituitary–adrenal axis, and with an alteration of bone metabolism due to prolonged dietary restrictions and excessive physical exercise. Implementation of eating disorder services as well as prevention strategies is necessary to avoid the deleterious effects of a long‐term DUI. Primary prevention would be directed at vulnerable subjects, for example, family members of patients with eating disorders, and would consist of clinical monitoring and possible psychosocial support (Jacobi et al. 2018). Secondary prevention would have as its objective the reduction of DUI and would consist of the creation of networks of healthcare professionals, internet‐based treatment guides, the creation of a specific outpatient clinic, and psychoeducation (short movies, posters) in the community and schools, as already proposed by some authors (Gumz et al. 2014).

Limits of the present study are (1) the retrospective collection of data, which in some cases implies the impossibility of tracing specific information on the recruited patients and introduces the risk of recall bias; (2) the fact that the data derive from a population belonging to specialized psychiatric services and, therefore, they are not always generalizable; (3) the collection of part of the clinical information during the peaks of the pandemic period which in itself could have negatively influenced the course of the disorder (Dumitrașcu et al. 2021); (4) the relatively small size of the sample, consisting only of female patients, which reduces the generalizability of the reported results; (5) some variables such as socioeconomic status, physical activity, and dietary habits, were not analyzed and might have affected the findings of the present article; (6) the cross‐sectional nature of the study, which does not allow to establish a cause–effect relationship; and (7) the presence of lifetime psychiatric comorbidity in about one‐third of patients could have affected DUI.

## Author Contributions

**Letizia Maria Affaticati**: conceptualization, data curation, formal analysis, investigation, methodology, writing – original draft. **Enrico Capuzzi**: conceptualization, data curation, formal analysis, investigation, methodology. **Tommaso Zuliani**: conceptualization, investigation, data curation. **Francesca Legnani**: data curation. **Nadia Vaccaro**: data curation. **Francesca Manzo**: data curation. **Alberto Scalia**: data curation. **Davide La Tegola**: data curation. **Monica Nicastro**: methodology. **Fabrizia Colmegna**: conceptualization. **Massimo Clerici**: conceptualization. **Antonios Dakanalis**: conceptualization, methodology. **Massimiliano Buoli**: conceptualization, methodology, data curation, formal analysis, investigation, writing – original draft, writing – review and editing.

## Conflicts of Interest

The authors declare no conflicts of interest.

### Peer Review

The peer review history for this article is available at https://publons.com/publon/10.1002/brb3.70584

## Data Availability

The data that support the findings of this study are not openly available due to reasons of sensitivity and are available from the corresponding author upon reasonable request.
